# Metal–Organic Framework‐Based Materials for Solar Water Splitting

**DOI:** 10.1002/smsc.202000074

**Published:** 2021-03-26

**Authors:** Xianlong Li, Zhiliang Wang, Lianzhou Wang

**Affiliations:** ^1^ Nanomaterials Centre School of Chemical Engineering and Australian Institute for Bioengineering and Nanotechnology The University of Queensland St. Lucia QLD 4072 Australia

**Keywords:** composite photocatalysts, metal–organic frameworks, photoelectrodes, solar-driven water splitting

## Abstract

Solar‐driven water splitting is a promising way to produce renewable hydrogen. The design of efficient semiconductor photocatalysts plays a vital role in this process. The recent dynamic development of metal–organic framework (MOF) materials provides a good opportunity to design efficient composite photocatalysts toward solar water splitting. Herein, a critical review of the design and development of MOF/semiconductor composite photocatalysts for solar‐driven water splitting is presented. The designing strategies of MOF‐based composite photocatalysts, including the use of organic ligands, quantum dots and carbon‐based materials to form heterostructures, and the combination of MOF layers with semiconductors to fabricate thin‐film photoelectrodes for photoelectrochemical water splitting, are introduced. The remaining challenges and future perspectives of the MOF‐based composites with the hope of achieving improved solar water splitting are also discussed.

## Graphical abstract

Metal–organic framework (MOF)‐based materials are promising candidates for solar water splitting because of their modifiable pores, abundant active sites, and tunable optical bandgap. By introducing organic ligands, combined with quantum dots and carbon to form composite photocatalysts, or coating MOF layer on semiconductor photoelectrodes, this article provides an overview of the MOF/semiconductor‐based photocatalysts towards efficient solar hydrogen production.

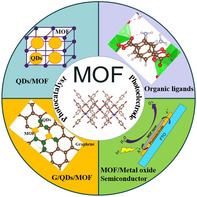

## 1 Introduction

The fast growing population and increasing industrial development have led to energy exhaustion and environmental issues.^[^ ^1^ ^]^ There is an urgent need to develop renewable and zero‐carbon emission systems as energy supply alternatives to the fossil fuels. Among various renewable options, such as hydroelectric, wind, tidal power, and solar energy,^[^ ^2^ ^]^ solar energy is regarded as the most attracting resource because of its abundance and cost‐effectiveness.^[^ ^3^ ^]^ Semiconductor‐based photocatalysts have the capability to convert solar energy into valuable chemicals such as hydrogen (H_2_) through a solar water splitting process, holding a good promise for powering our society with green hydrogen.^[^ ^4^ ^]^

One important milestone in solar water splitting is the discovery of unbiased hydrogen evolution reaction (HER) under UV light using a TiO_2_ photoelectrode.^[^ ^5^ ^]^ Later on, Khaselev and Turner were able to achieve a decent solar‐to‐hydrogen (STH) conversion efficiency of 12.4% by applying effective III–V semiconductors as the photoelectrode materials.^[^ ^6^ ^]^ It highlights the potential of solar water splitting for H_2_ production. In comparison with the water electrolysis process for H_2_ production, a photocatalytic water splitting system is advantageous in the terms of energy consumption, system simplicity, scalability, and cost. Thus, the development of sustainable solar‐driven water splitting represents an important technological breakthrough for the renewable energy supply.

To achieve sustainable large‐scale hydrogen production, the photocatalysts should be cheap, abundant, and less toxic. In this regard, some material candidates, such as TiO_2_, g‐C_3_N_4_, and Cu_2_O, are highly sought‐after for solar‐driven water splitting.^[^ ^7^ ^]^ However, their intrinsic properties, including wide bandgap, low charge mobility, fast charge carrier recombination, etc., have limited their STH efficiency.^[^ ^8^ ^]^ To overcome the challenges in these photocatalysts, the design of composite photocatalysts by combining the advantages of different functional components is an option.^[7b,e]^ The composite photocatalysts are expected to improve the properties of three key steps in solar water splitting, including light harvesting, charge separation and transfer, and surface reaction, which are not easily achieved in a single semiconductor.^[^ ^9^ ^]^

Metal–organic framework (MOF) materials have attracted increasing attention due to their advantages of controllable porous size and tunable framework structure and compositions. By choosing suitable central metal atoms, it is possible to create photoresponsive MOF materials. Moreover, the large surface areas and selective porosities in MOF materials can be potentially applied in gas–solid phase reactions, such as CO_2_ reduction reaction.^[^ ^10^ ^]^ Thus, the combination of MOFs with appropriate semiconductors can be a promising approach to design effective solar conversion processes,^[^ ^11^ ^]^ whereas a comprehensive overview on this new type of MOF/semiconductor photocatalysts in water splitting is still lacking.

In this review, we revisit the recent progress of MOF/semiconductor composite photocatalysts for solar water splitting. The mechanism of solar water splitting based on photocatalysis (PC) and photoelectrocatalysis (PEC) is briefly introduced. The design of MOF/semiconductor composite photocatalysts for PC and PEC is discussed in detail. To further promote the progress of this emerging research field, the current challenges and future outlook of MOF/semiconductor composite photocatalysts for solar hydrogen production are also presented.

## 2 Mechanisms of Solar Water Splitting Reactions

In general, the solar conversion process on a photocatalyst involves three basic steps as aforementioned, light harvesting, charge separation and transfer, and the subsequent surface reaction.^[^ ^12^ ^]^ When a semiconductor is stimulated by sunlight, electrons in the valence band (VB) can be excited to the conduction band (CB), leaving photogenerated holes in VB. The bandgap of semiconductor determines the utmost light harvesting capability for solar water splitting.^[^ ^13^ ^]^ The photogenerated electron–hole pairs are required to separate and migrate to the semiconductor/solution interface. During this process, the charge carriers normally suffer from serious recombination.^[^ ^6^, ^14^ ^]^ Therefore, suppressing the recombination process is a priority in achieving an efficient solar conversion process. For the survived photogenerated electrons that reach surface reaction sites, the surface reaction will take place. With appropriate selection of cocatalysts, the surface reaction can be further accelerated, leading to improved solar conversion efficiency.

**Figure 1:**
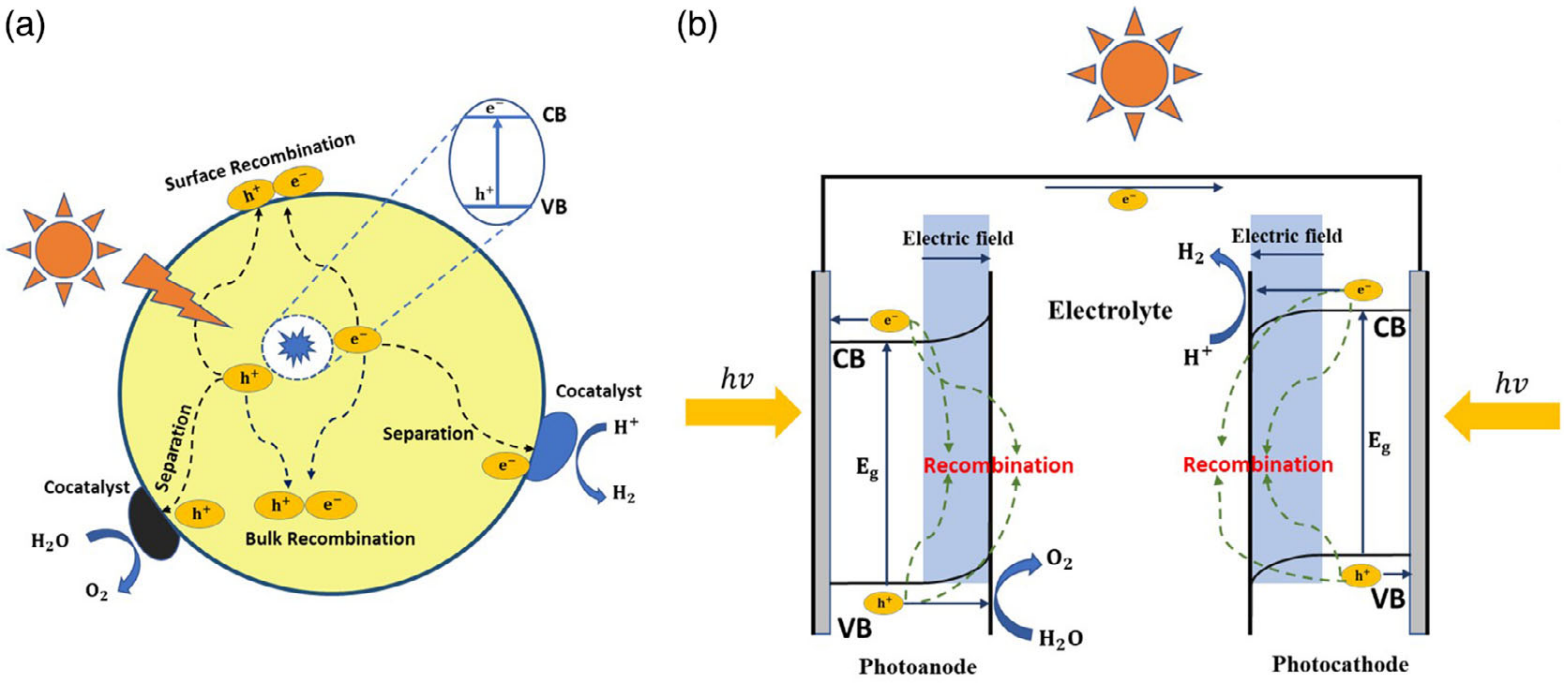
Schematic illustrations of a) the PC and b) the PEC water splitting processes.

There are two paradigms to achieve direct STH conversion process, i.e., the PC and PEC. The driving force for charge separation and transfer in both PC and PEC processes originates from band bending at the semiconductor–electrolyte interface, where a built‐in electric field (*E*_bi_) can be established (**Figure** **1**a).[^8^, ^14^] Specifically, in the PEC process, the degree of band bending can be adjusted by applied bias. In a dual‐photoelectrode system, as shown in Figure 1b, the upward band bending on the photoanode drives the photogenerated holes toward the photoelectrode–electrolyte interface.^[^ ^14^ ^]^ While on the photocathode, the photogenerated electrons will transfer to the active sites by downward band bending.^[^ ^15^ ^]^ A good combination of the two photoelectrodes with proper band alignment to allow efficient charge separate and transport is the key to realize good PEC water splitting performance.

## 3 Structures and Properties of MOF Materials

MOF materials are a class of microporous crystal complexes composed of metal ions/clusters coordinating with organic ligands.^[^ ^16^ ^]^ MOF materials show a tunable topological structure with large surface areas and highly ordered pores, which can be used for accumulating reactants.[^7^, ^16^] The property of MOF materials can be tailored by adjusting the central metal atoms and organic linkers. An appropriate hydrophilic or hydrophobic pore property in some MOF materials is useful to enhance the adsorption and recognition of key reactant and product molecules.^[^ ^17^ ^]^ Therefore, MOF materials have been applied in a broad range of applications, including gas separation/storage, photo‐driven water splitting,^[^ ^18^ ^]^ carbon dioxide reduction reaction,[^1^, ^10^, ^19^] organic pollutant degradation,[^4^, ^16^, ^20^] supercapacitors,^[^ ^21^ ^]^ biosensing,^[18a]^ nanomedicine,^[^ ^22^ ^]^ and sensing device fabrication.^[^ ^22^, ^23^ ^]^ The first visible‐light responsive MOF composite photocatalyst was reported in 2009.^[^ ^24^ ^]^ Since then, increasing attention has been paid to the MOF‐based solar water splitting. However, intrinsic drawbacks of pure MOF materials, including weak light absorption, low conductivity, instability, and rapid charge recombination, are the major obstacles toward efficient solar hydrogen production. For example, Zr‐based MOF materials (e.g., zeolitic imidazolate framework (ZIF)) can only absorb UV light (~5% of sunlight) due to their wide bandgap, leading to low efficiency.[^7^, ^25^] More effort is required to improve the physiochemical properties of MOF materials to achieve higher solar conversion efficiency.

## 4 MOF‐Based Materials Applied in PC

MOF crystals can be directly used as powder photocatalysts in the PC water splitting. The rich porous structures of MOF materials provide a good host to immobilize other components, such as nanosized semiconductors or photosensitizers.^[^ ^26^ ^]^ The surface modification using linkers, such as the addition of functional groups, can also lead to improved optoelectric properties. Many interesting studies have been reported in fabricating MOF‐based composites photocatalysts, whereas some of them demonstrated half reactions of hydrogen production in the presence of sacrificial agents, as shown in **Table** **1**. In the following sections, we will introduce several groups of MOF‐based composite photocatalysts.

**Table 1:** MOF‐based composite photocatalysts for the PC water splitting

Photocatalyst	*E*_g_ [eV]	Sacrificial agents	Photosensitizers	Cocatalyst	H_2_ production rate [μmol h^−1^]			Ref.
MoS_2_ QDs/UiO‐66‐NH_2_/Graphene oxide	–	–	Eosin Y	MoS_2_		62.12	[35]	
MoS_2_/UiO‐66/CdS	–	–	–	MoS_2_		650	[33]	
CdS@MIL‐101(Cr)	–	Lactic acid	–	Carbon dot (CD)		14.66	[34]	
CdS@NU‐1000/Reduced graphene oxide (RGO)	–	0.1 m S^2−^ +0.1 m SO_3_^2−^	–	Pt		12.1	[48]	
UiO‐66/CdS/RGO	–	0.1 m SO_3_^2−^ +0.1 m S^2−^	–	Pt		13.8	[49]	
UiOS‐Cu‐CdS/ZnS	2.64	0.35 m S^2−^ +0.25 m SO_3_^2−^	–	–		425.5	[29]	
CdS@ZAVCl MOF	3.9	–	–	CdS		41.8	[32]	
Cd(OH)_2_/CdS—C_3_N_4_	2.32	Lactic acid	–	Cd(OH)_2_		148.13	[38]	
g‐C_3_N_4_	–	Triethanolamine	–	NiCoP_2_		42.89	[37]	
MIL‐101	–	–	Erythrosin B dye	Ni/NiO_*x*_		125	[16c]	
UiO‐66/g‐C_3_N_4_	–	Ascorbic acid	–	Pt		14.11	[36]	

### 4.1 Organic Functional Group Modification

**Figure 2:**
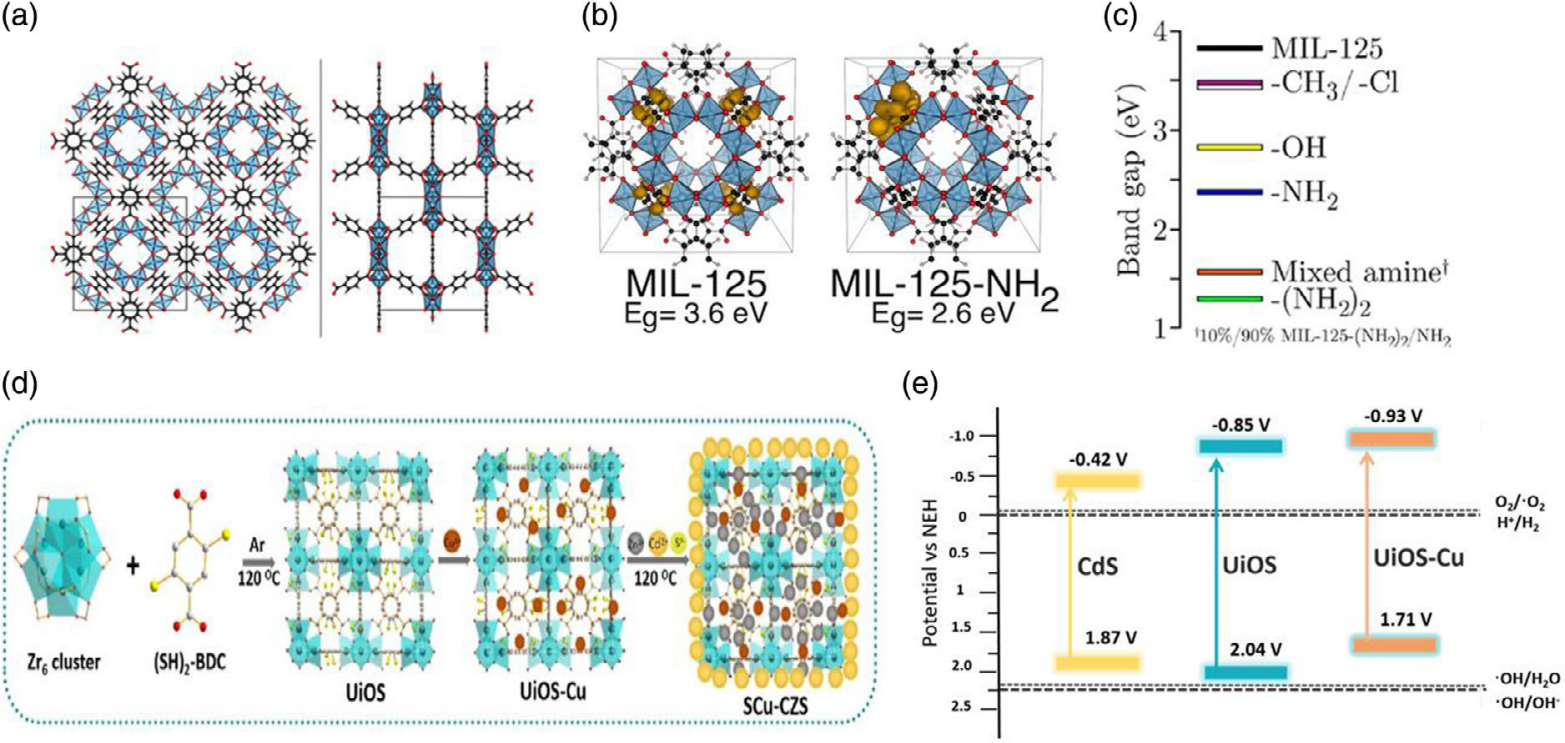
a) The structure and b) Frontier electron density of the VB of MIL‐125 and MIL‐125‐NH_2_. c) Bandgap of MIL‐125‐R (—(NH_2_)_2_, —NH_2_, —OH, —CH_3_, and —Cl). a–c) Reproduced with permission.^[^ ^28^ ^]^ Copyright 2013, American Chemical Society. d) Schematic synthesis and e) mechanism for the photocatalytic process of SCu‐CZS. d,e) Reproduced with permission.^[^ ^29^ ^]^ Copyright 2020, Elsevier.

Introducing organic functional groups with high polarity has been reported to reduce the bandgap of the MOFs.^[^ ^27^ ^]^ For instance, **Figure** **2**a–c shows that Ti‐based MOF, MIL‐125, composed of TiO_2_ unit and organic functional groups BDC‐R (1,4‐benzenedicarboxylic (BDC) and R is the functional group) can be designed with different functional groups, including —(NH_2_)_2_, —NH_2_, —OH, —CH_3_, and —Cl.^[^ ^28^ ^]^ Due to the electron donation from N 2p orbital to the aromatic linker, the VB of MIL‐125‐NH_2_ is modified (Figure 2b), and the optical bandgap shifts to the visible light region (≈2.6 eV/475 nm).^[^ ^28^ ^]^ Theoretical investigation indicated that the —(NH_2_)_2_ groups can improve the electron density, leading to a decreased bandgap (1.55 eV), as shown in Figure 2c.^[^ ^28^ ^]^ In another example, a new Zr‐based MOF composite photocatalyst is prepared by combining UiO‐66 with organic functional groups for PC water splitting.^[^ ^29^ ^]^ The —SH groups are applied to modify the BDC linker to form the UiOS, as indicated in Figure 2d. Then, Cu ions are anchored onto the UiOS framework because of the coordination of Cu—S bonds. The —SH modification obviously reduces the bandgap of UiOS due to the elevated VB edge compared with that of the unmodified UiO‐66 (Figure 2e).^[^ ^30^ ^]^ The abovementioned examples suggest the significance of organic functional groups in adjusting the optoelectric properties of the MOF materials, providing a good strategy in modifying the bandgap in a controllable manner. Based on the band theory of solids, after such modification, some MOF materials may demonstrate semiconducting property, thereby increasing the likelihood of MOF‐based composites for solar‐driven water splitting. The abovementioned results clearly indicates the effectiveness of functional adjustment of the bandgap. However, these functional groups are normally highly reactive, which may deteriorate the stability of MOF materials. Thus, how to achieve a good balance of photocatalytic performance and stability of the MOF materials requires further research.

### 4.2 Quantum Dot Modification

**Figure 3:**
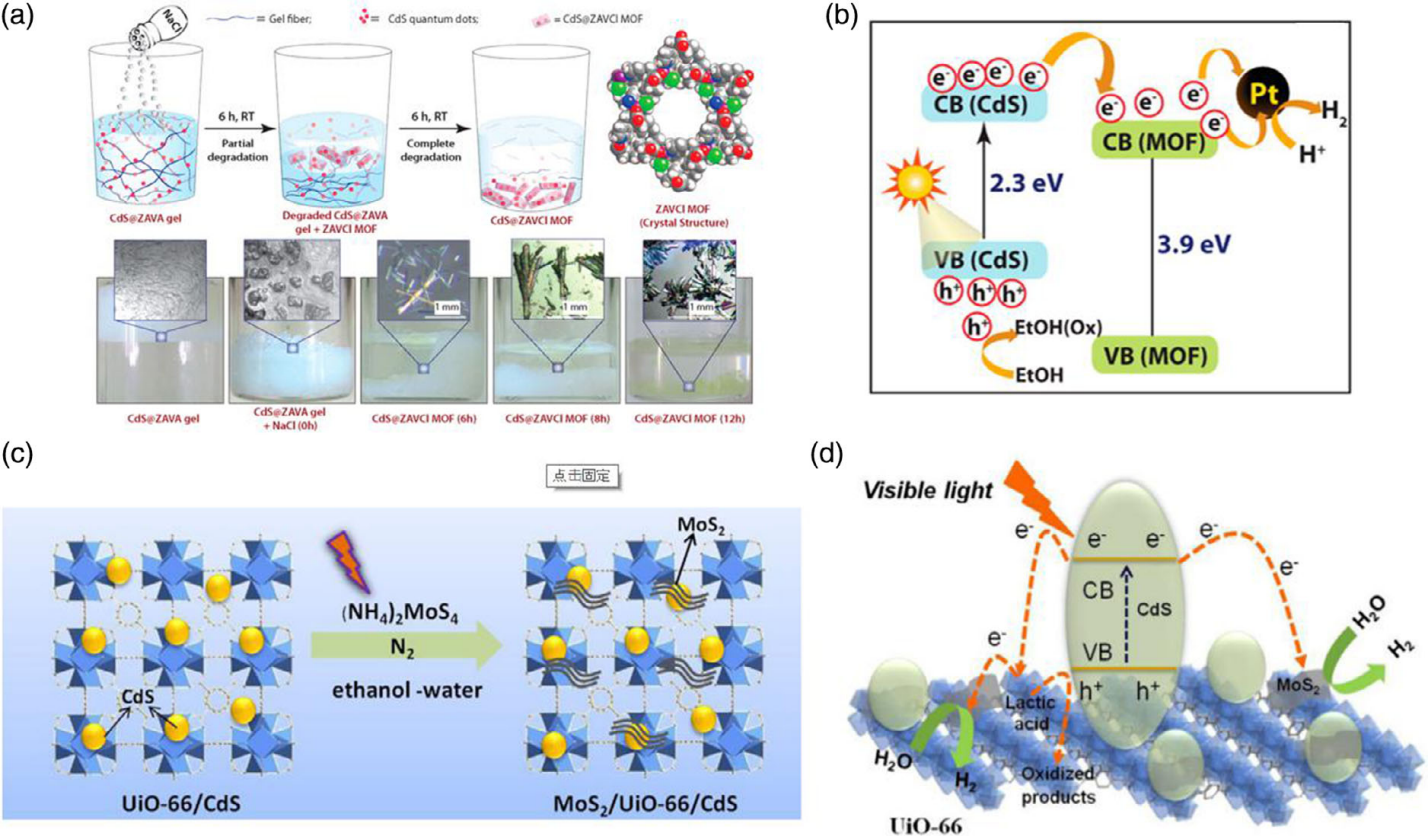
a) Schematic synthesis and b) mechanism for the photocatalytic process of CdS@ZAVA‐MOF. a,b) Reproduced with permission.^[^ ^32^ ^]^ Copyright 2014, American Chemical Society. c) Schematic synthesis and d) mechanism for the photocatalytic process of MoS_2_/UiO‐66/CdS. c,d) Reproduced with permission.^[^ ^33^ ^]^ Copyright 2014, Elsevier.

Quantum dots (QDs) as a class of important functional materials have specific physicochemical properties, such as narrow photoluminescence emission spectrum, long carrier lifetimes, and high photoluminescence quantum yield.[^10^, ^16^, ^31^] Because of the small size of QDs, the porous structure of MOFs can act as a good host to accommodate functional QDs. By introducing CdS QDs into Zn (II)‐based MOF, ZAVA (ZAVA: Zn(II)‐based low‐molecular‐weight metallohydrogel), a class of CdS@ZAVA‐MOF composite photocatalyst can be prepared through a bottle‐around‐the‐ship method (**Figure** **3**a).^[^ ^32^ ^]^ It is reported that the CdS QDs can form heterojunction with the ZAVA‐MOF, which facilitates the charge separation in this composite photocatalyst, as shown in Figure 3b. By taking advantage of abundant anchoring sites of the UiO‐66 structure, the QDs of both CdS and MoS_2_ can be loaded on the host materials to form a new type of Zr‐based MOF‐QDs composite photocatalysts of MoS_2_/UiO‐66/CdS (Figure 3c).^[^ ^33^ ^]^ The H_2_ production rate on this composite sample outperforms that of pure CdS and Pt/UiO‐66/CdS photocatalysts under the same reaction conditions, which may be attributable to not only the synergistic catalytic effect within each component in MoS_2_/UiO‐66/CdS, but also the enriched active sites provided by MoS_2_ QDs (Figure 3d). Meng et al. developed a type of CQDs/CdS@MIL‐101 (CQDs: carbon QDs) materials through a one‐step double solvents method.^[^ ^34^ ^]^ The improved photocatalytic performance of this carbon‐modified ternary photocatalytic system is associated with the unique role of CQDs in accelerating the electron mobility and suppressing the photogenerated electron/hole pair recombination. These examples show the high potential of functional QDs in solar water splitting, yet the controllable and homogeneous anchoring of the QD functional components onto the MOF structure remains a challenge.

### 4.3 Carbon Modification

**Figure 4:**
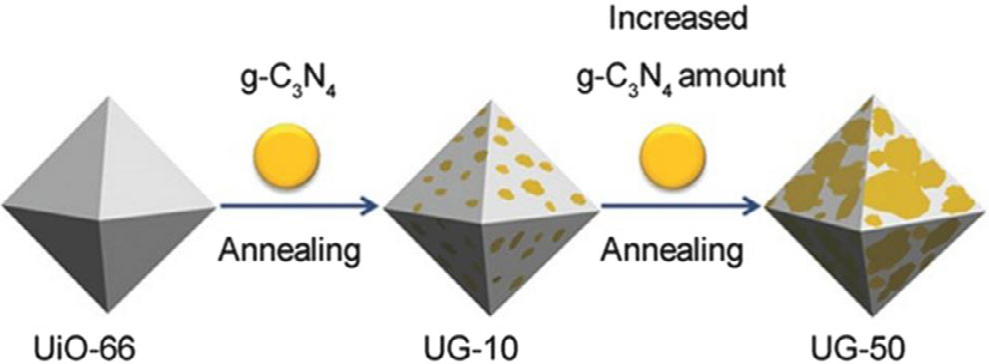
Schematic synthesis of UiO‐66/g‐C_3_N_4_ photocatalyst. Reproduced with permission.^[^ ^36^ ^]^ Copyright 2015, Wiley‐VCH.

Carbon‐based materials such as graphene have the advantage of excellent conductivity to facilitate the photogenerated electron–hole pairs, providing a good material platform to design the MOF‐based composite photocatalysts.^[19a]^ An interesting example is the use of graphene to prepare MoS_2_/UiO‐66‐NH_2_/G (G: graphene) ternary composites through the “bottle‐around‐the‐ship” method.^[^ ^35^ ^]^ The resulting composites exhibited excellent photocatalytic performance because of the synergistic effect of the each component, in which the MOF provides porous structure for Eosin dye adsorption and graphene ensures fast electron mobility. In addition to graphene, other materials, e.g., graphitic C_3_N_4_, have also been applied in the MOF composite photocatalyst fabrication. For example, Wang et al. prepared a class of UiO‐66/g‐C_3_N_4_ binary composite materials through a thermal annealing method for photocatalytic water splitting (**Figure** **4**).^[^ ^36^ ^]^ Systematic study revealed that the improved performance can be attributed to the increased light‐harvesting capability of g‐C_3_N_4_ and accelerated interfacial charge mobility from g‐C_3_N_4_ to MOF. The abovementioned examples provide good demonstration of carbon‐decorated MOF materials for PC. However, the light screening effect from the carbon materials will be a major concern in designing effective photocatalyst, which needs further research in the future.

### 4.4 MOF‐Derived Carbon Materials

**Figure 5:**
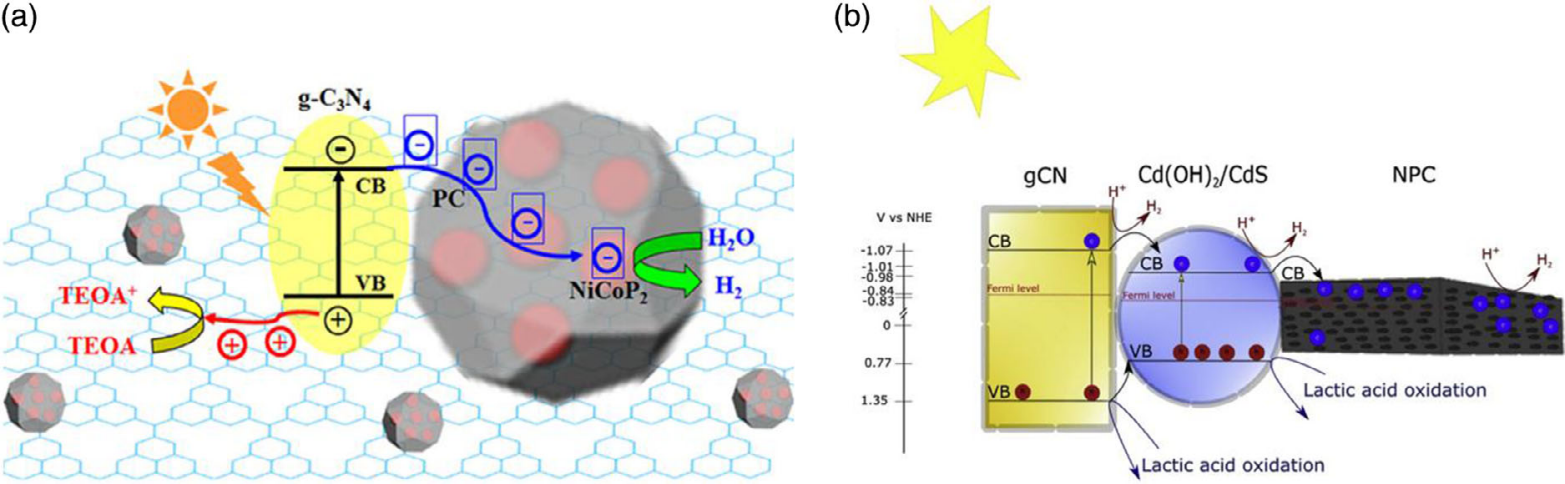
Mechanism for the photocatalytic process of a) g‐C_3_N_4_/NiCoP_2_‐PC. Reproduced with permission.^[^ ^37^ ^]^ Copyright 2019, American Chemical Society. b) CDs/CdS@MIL‐101. Reproduced with permission.^[^ ^38^ ^]^ Copyright 2019, Elsevier.

MOF materials have versatile functionalities and can also act as sacrificial templates to produce nanoporous carbon (NPC)‐based composites for water splitting purpose. A typical example is the design of g‐C_3_N_4_/NiCoP_2_/porous carbon composite photocatalysts by a thermal treatment of NiCo‐MOF.^[^ ^37^ ^]^ The composite photocatalyst inherits the similar porous structure of the MOF template, which minimizes the risk of g‐C_3_N_4_ stacking. A remarkably improved photocatalytic H_2_ production rate is achieved due to the accelerated charge transfer and decreased overpotential of HER (**Figure** **5**a). In another example, Aleksandrzak et al. fabricated CdS/g‐C_3_N_4_‐NPC composite photocatalyst by carbonizing Al‐MOF (Figure 5b).^[^ ^38^ ^]^ This MOF‐based carbon material plays vital role in reducing the reaction barrier of HER and boosting the charge mobility and separation. These studies illustrate the potential of MOF materials as precursors and/or templates to synthesize cost‐effective carbon‐based photocatalysts, which normally feature some unique nanostructures, such as high porosity and high specific surface area for satisfying the requirement of water splitting photocatalysts.

## 5 MOF‐Based Materials for PEC

The fabrication of high‐quality photoelectrodes is a prerequisite for PEC water splitting. The intimate contact between the conductive substrates and active semiconductor is important for quality photoelectrode fabrication. For MOF‐based semiconductors, there is still a challenge to form strong contact with conductive substrates such as fluorine‐ or indium‐doped tin oxide (FTO or ITO) due to the weak interaction between the MOF materials and the substrates. Alternatively, the substrate can be coated with a precursor semiconductor layer, which will be used for the deposition MOF layer. Furthermore, MOFs can be used as sacrificial templates for fabricating MOF‐derived semiconductors. In the following section, we will briefly discuss different MOF (Ti‐based, Fe‐based, Zn‐based, and Co‐based) composite photoelectrodes applied in PEC water splitting (shown in **Table** **2**).

**Table 2:** MOF‐based composite photoelectrodes for PEC water splitting

Photoelectrode	Metal unit		Cocatalyst	Light source	Electrolyte	*J* [mA cm^−2^]	Ref.
BiVO_4_@NiFe‐MOFs	Ni/Fe	–		AM 1.5G lamp	0.5 m KBi + 0.05 m V_2_O_5_	5.3 (at 1.23 V_RHE_)	[47]
M‐TiO_2_/CdSe@CdS QDs	Ti	CdS		AM 1.5G lamp	0.25 m Na_2_S + 0.35 m Na_2_SO_3_	10.72 (at 0.9 V_RHE_)	[40]
ZnO@MOF‐derived ZnS/CoS	Zn	MOF‐derived ZnS/CoS		Xenon lamp	0.5 m Na_2_SO_4_	2.46 (at 0.6 V_SCE_)	[44]
Fe_2_O_3_:Ti/NH_2_‐MIL‐101	Fe	–		AM 1.5G lamp	1 m NaOH	2.27 (at 1.23 V_RHE_)	[50]
CoNi‐MOF/BiVO_4_	Co/Ni	CoNi‐MOF		AM 1.5G lamp	0.5 m Na_2_SO_4_	3.2 (at 1.23 V_RHE_)	[51]
MOF‐derived Co_3_O_4_/BiVO_4_	Co	–		Xenon lamp	0.5 m KH_2_PO_4_	2.35 (at 1.23 V_RHE_)	[52]
Mo:BiVO_4_–MIL‐53	Fe	–		AM 1.5G lamp	0.2 m Na_2_SO_4_	2.2 (at 1.23 V_RHE_)	[4c]
Ag/NH_2_‐MIL‐125/TiO_2_	Ti	Ag		Xe lamp	0.5 m Na_2_SO_4_	1.06 (at 1.23 V_RHE_)	[53]
BiVO_4_@Co‐MIm	Co	Co‐MIm		AM 1.5G lamp	0.5 m Na_2_SO_4_	3.16 (at 1.23 V_RHE_)	[46]
Ti_*x*_Fe_1‐*x*_O_*y*_/Fe_2_O_3_	Ti	–		AM 1.5G lamp	1 m NaOH	0.724 (at 1.23 V_RHE_)	[54]
Fe/W co‐doped BiVO_4_/MIL‐100	Fe	MIL‐100		Xe lamp	0.1 m Na_2_SO_4_	2.76 (at 1.23 V_SCE_)	[41]
Cobim/BiVO_4_	Co	Cobim		AM 1.5G lamp	0.5 m Na_2_SO_4_ + 0.2 m Na_2_SO_3_	3.1 (at 1.23 V_RHE_)	[45]
ZnNi MOF@ZnO	Zn/Ni	ZnNi MOF		AM 1.5G lamp	0.5 m Na_2_SO_4_	1.40 (at 1.23 V_RHE_)	[42]
ZnO@Au@ZIF‐67	Zn	–		Xe lamp	0.5 m Na_2_SO_4_	1.93 (at 0.6 V_SCE_)	[55]
TNAs@Ti‐MOF	Ti	–		Xenon lamp	0.42 m NaCl +0.029 m Na_2_SO_4_	3.04 (at 1.23 V_RHE_)	[23]

### 5.1 Ti‐Based MOF Composite Photoelectrodes

**Figure 6:**
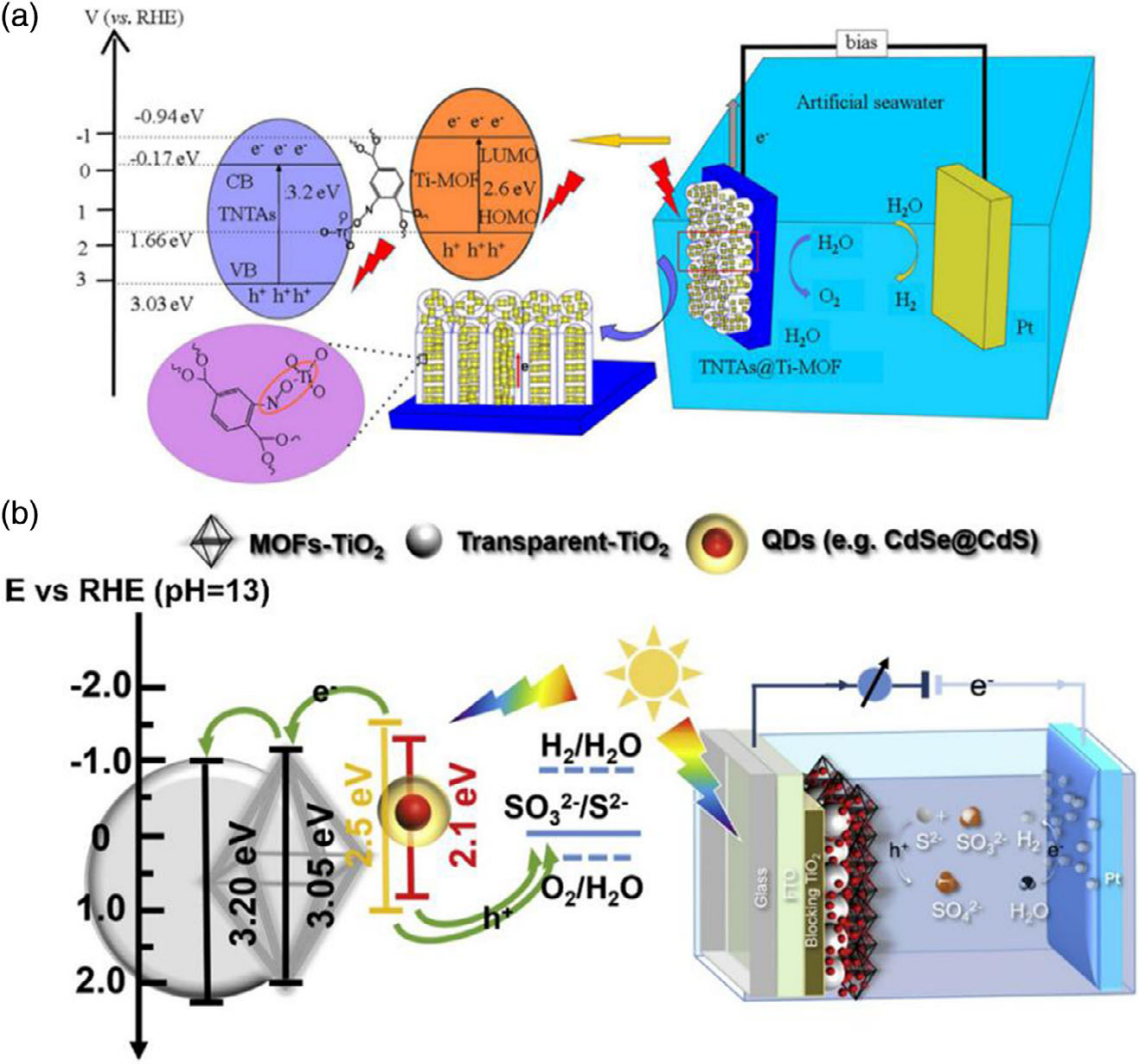
Schematic illustration of the mechanism of the PEC on the heterostructures of a) TNAs@Ti‐MOF composite photoelectrode. Reproduced with permission.^[^ ^23^ ^]^ Copyright 2019, Elsevier. b) (M‐TiO_2_/QDs)/QDs photoanode. Reproduced with permission.^[^ ^40^ ^]^ Copyright 2019, Elsevier.

The TiO_2_ nanotube arrays (TNAs) are frequently applied for photoelectrode fabrication, whereas Ti‐based MOFs normally exhibit considerable photoresponse. Inspiring by these features, researchers have put effort in designing Ti‐MOF/TiO_2_ composite semiconductors for the PEC process. One example is to grow Ti‐based MOF using TNA layer as the Ti source through a hydrothermal process, as shown in **Figure** **6**a.^[^ ^23^ ^]^ The heterojunction formed between TiO_2_ and Ti‐MOF can promote charge separation in TNAs@Ti‐MOF composite photoelectrode. After modifying Ti‐MOF with functional group of —NH_2_, the light‐absorption region is extended.^[^ ^23^, ^39^ ^]^ Ti‐based MOF can also be used as a template for TiO_2_ synthesis. Benetti and co‐workers successfully fabricated an octahedral‐structure TiO_2_ with anatase–rutile–mixed phase form MIL‐125‐NH_2_.^[^ ^40^ ^]^ After loading with CdSe@CdS QDs (Figure 6b), the photocurrent of TiO_2_ photoanode (M‐TiO_2_/QDs) shows over 50% improvement compared with the anatase TiO_2_ photoanode.^[^ ^40^ ^]^ However, for these systems, the stability is still a challenge due to the easy decomposition of organic ligands, which requires careful modification and better material selection.

### 5.2 Fe‐Based MOF Composite Photoelectrodes

**Figure 7:**
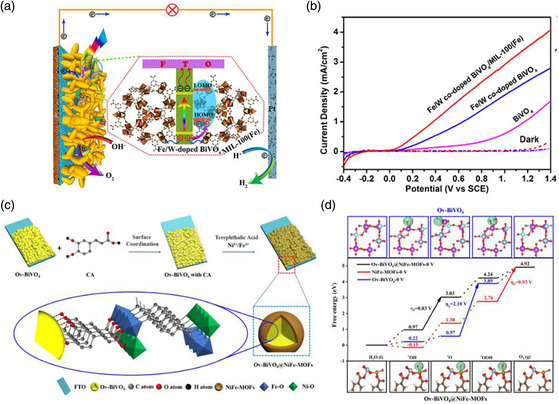
a) Proposed charge transfer mechanism over the heterostructures and b) PEC test results of Fe/W co‐doped BiVO_4_ photoelectrode. a,b) Reproduced with permission.^[^ ^41^ ^]^ Copyright 2016, Wiley‐VCH. c) Schematic illustration of the synthesis and d) free energy diagram for OER pathway on core–shell Ov‐BiVO_4_@NiFe‐MOFs. c,d) Reproduced with permission.^[^ ^47^ ^]^ Copyright 2020, Wiley‐VCH.

Fe‐based MOF materials have a narrow bandgap with strong visible light absorption, which normally exhibit promising activity for oxygen evolution reaction (OER). Thus, this group of Fe‐containing materials have been applied as the cocatalysts in photoelectrode design. For example, Jiao et al. modified Fe/W co‐doped BiVO_4_ photoelectrode with Fe‐based MOF, MIL‐100(Fe), for PEC water splitting (**Figure** **7**a),^[^ ^41^ ^]^ and achieved 14 times higher photocurrent than that of pure BiVO_4_ (Figure 7b).^[^ ^41^ ^]^ Pan et al. developed a new type of BiVO_4_ photoanodes with NiFe‐MOF loaded on the surface, as shown in Figure 7c. The density functional theory calculations revealed that the NiFe‐MOFs coating could decrease the overpotential for OER (Figure 7d), thus improving the photocurrent density and decreasing the onset potential on the photoelectrodes.

### 5.3 Zn‐Based MOF Composite Photoelectrodes

**Figure 8:**
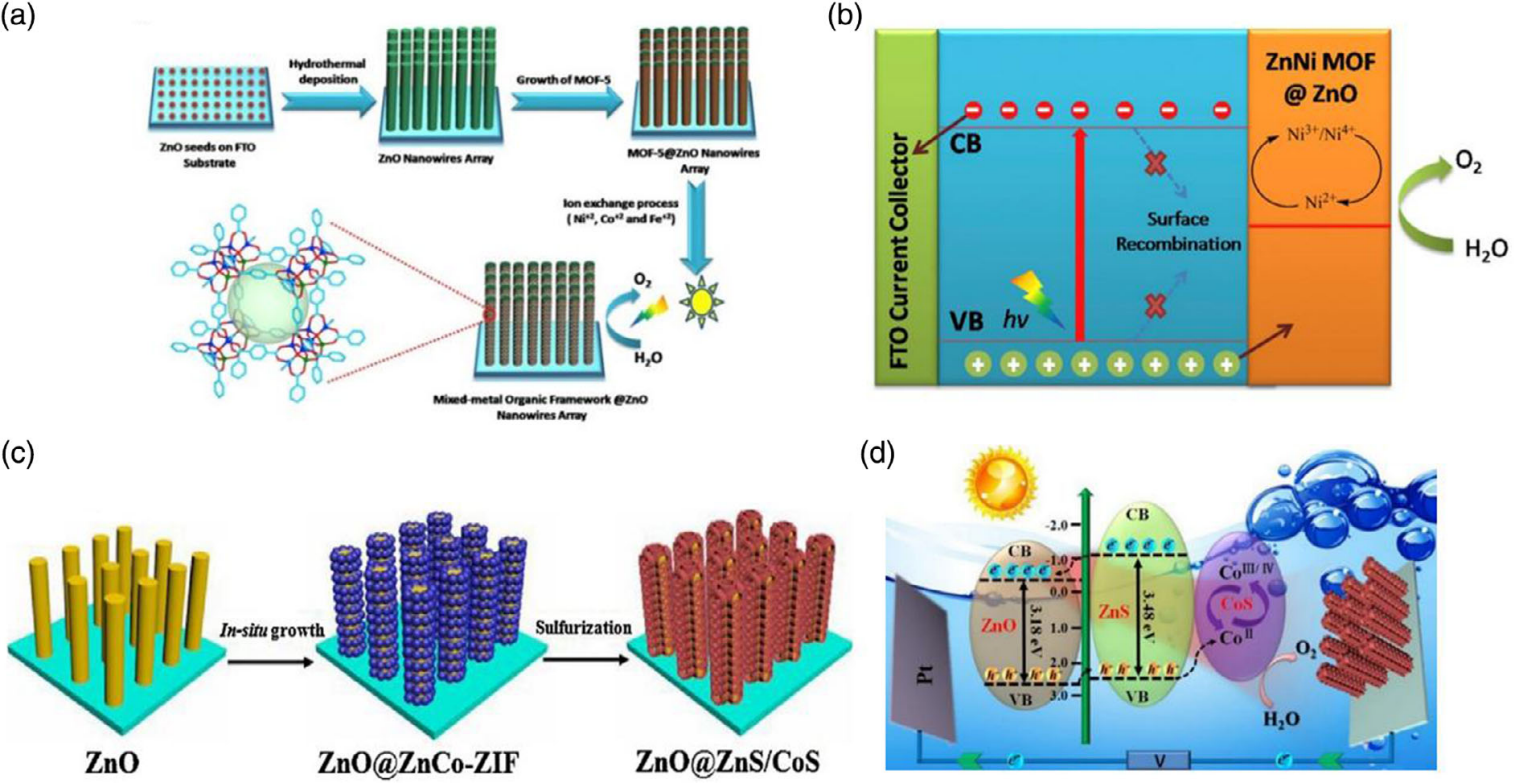
a) Schematic illustration of the synthesis procedure and b) charge transfer mechanism on the heterostructure of ZnNi‐MOF@ZnO composite photoelectrodes. a,b) Reproduced with permission.^[^ ^42^ ^]^ Copyright 2018, Hydrogen Energy Publications LLC. Published by Elsevier. c) Schematic synthesis and d) the proposed charge transfer mechanism over the heterostructure of ZnO@ZnS/CoS MOF‐derived photoelectrode. c,d) Reproduced with permission.^[^ ^44^ ^]^ Copyright 2017, Elsevier.

Zn‐based MOF materials have been applied in PEC research due to their capability to form heterostructure for improved charge separation.^[^ ^42^, ^43^ ^]^ For example, Peng et al. fabricated Zn and Ni mixed‐metal MOF‐coated ZnO nanowire array (ZnNi‐MOF@ZnO) photoanode through ion‐exchange processing, as shown in **Figure** **8**a.^[^ ^42^ ^]^ The covalent interactions between ZnO and MOF may create intimate interfacial contact, which accelerates charge transfer to the reaction interface under illumination. Zhou et al. fabricated ZnO@ZnS/CoS photoelectrode through sulfurization of ZnCo‐ZIF coated ZnO arrays (Figure 7c).^[^ ^44^ ^]^ This strategy not only forms heterojunction with the unique cellular structure for rapid electron/hole pair separation (Figure 7d), but offers rich exposed active sites for boosting reaction kinetics. These studies indicate that using inorganic semiconductor substrate as the precursor source could provide a good approach for the in situ formation of MOFs as the cocatalyst, thereby forming strong interaction between the MOF materials and the inorganic semiconductor substrate for efficient PEC water splitting.

### 5.4 Co‐Based MOF Composite Photoelectrodes

**Figure 9:**
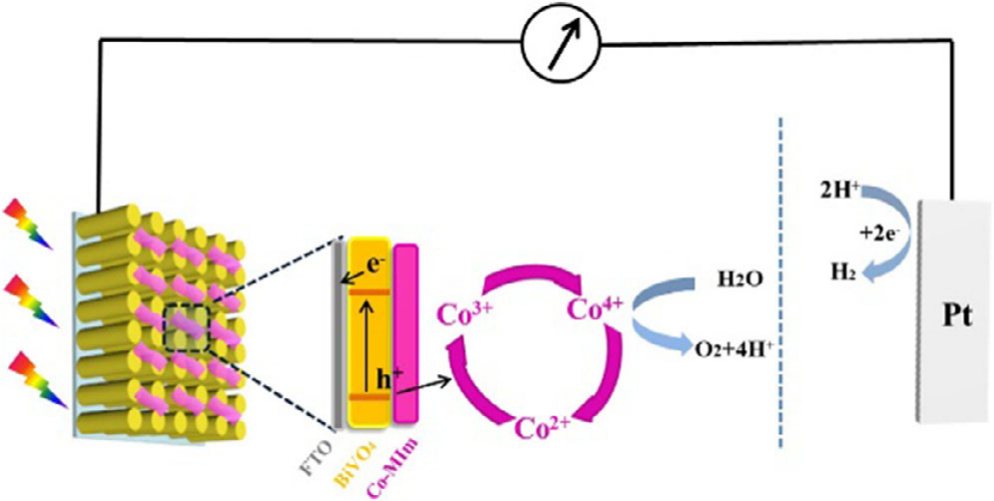
Proposed charge transfer mechanism over the heterostructure of Co‐MIm/BiVO_4_ photoelectrode. Reproduced with permission.^[^ ^46^ ^]^ Copyright 2019, Elsevier.

In addition to the abovementioned transition metal‐based MOF composites, Co‐containing MOF materials have also been a research focus because of their excellent performance toward oxygen‐involved electrocatalytic reactions, as a result of the physical/chemical properties of small particle size, high porosity, and abundant metal sites of the MOF materials. Zhang et al. fabricated a Co‐MOF‐modified BiVO_4_ photoanode through ultrathin sheet‐induced growth strategy.^[^ ^45^ ^]^ The Co‐MOF/BiVO_4_ photoanode showed better photocurrent density than the original or CoO_*x*_‐modified BiVO_4_ photoanode. Other kinds of Co‐based MOFs, (Co‐MIm) nanosheets, were applied as the cocatalysts to modify BiVO_4_ photoanode for PEC water splitting (**Figure** **9**),^[^ ^46^ ^]^ suggesting the good potential of Co‐based MOF layers as cocatalyst on MOF composite photoelectrodes.

Despite numerous examples of MOF/semiconductor composite photoelectrodes, their PEC performance is still relatively low. The MOF‐based composite photoelectrodes have been mainly limited to abovementioned groups such as Ti (MIL‐125), Fe (e.g., MIL‐100/), Zn (e.g., ZIF‐8), and Co (e.g., Co‐MIm)‐based materials to date. It is urgently required to explore and develop a wider range of efficient metal‐based MOF photoelectrodes to further improve the key parameters such as higher photocurrent density and lower the onset potential to achieve sustainable PEC water splitting.

## 6 Conclusion and Outlook

Solar water splitting is a promising strategy for capturing and converting renewable solar energy to hydrogen fuel sustainably. This review has analyzed the recent progress in MOF/semiconductor composite photocatalysts for solar water splitting. For powder‐based PC, the modification of MOF materials using organic functional ligands has been demonstrated to be an effective method. Other functional components such as QDs, graphene, and nanosized semiconductor particles have also been used to satisfy the solar water splitting requirement in the MOF‐based composites. While for the film‐based PEC system, the recent progress of MOF coating with transition metal oxide semiconductors in the aspects of photoelectrode fabrication, PEC performance improvement was overviewed briefly. Despite some progress on MOF‐based composite as photocatalyst/photoelectrode, more significant efforts should be considered to overcome the following challenges.

Intrinsically, many MOF materials have low light harvesting capability, which is a main bottleneck for achieving high photocatalytic performance. The soft structure of MOF can be collapsed during the solar water splitting reaction, raising the alarm on stability concern of MOF materials. In addition, the intrinsic poor conductivity of MOF materials limits the charge carrier separation and transfer.

To address these challenges, the following strategies can be considered. First, it is important to explore and develop more conductive MOF materials to promote the electron–hole separation and transfer in both the PC and PEC systems. In‐depth understanding of the charge transfer between the interfaces of MOF/semiconductor composites is significant for designing MOF‐based solar‐driven water splitting. Advanced characterization tools such as in situ spectroscopic and structural observations of the charge transfer, reaction intermediates, and products can provide useful information. Furthermore, it is necessary to combine experimental and theoretical approaches to clarify the ambiguous water splitting mechanism on MOF‐based materials. The advances of machine learning in catalyst design and selection may also power the development of new generation of MOF‐based composites to satisfy the sustainable hydrogen production demand in the not distant future.

## Conflict of Interest

The authors declare no conflict of interest.

## References

[1] a) X. Deng , Y. Qin , M. Hao , Z. Li , Inorg. Chem. 2019, 58, 16574; 31774657 10.1021/acs.inorgchem.9b02593

[2] a) N. S. Lewis , D. G. Nocera , Proc. Natl. Acad. Sci. USA 2006, 103, 15729; 17043226 10.1073/pnas.0603395103 PMC1635072

[3] a) X. Xiong , C. You , Z. Liu , A. M. Asiri , X. Sun , ACS Sustain. Chem. Eng. 2018, 6, 2883;

[4] a) L. Pan , S. Wang , J. Xie , L. Wang , X. Zhang , J.-J. Zou , Nano Energy 2016, 28, 296;

[5] a) B. Weng , M.-Y. Qi , C. Han , Z.-R. Tang , Y.-J. Xu , ACS Catal. 2019, 9, 4642;

[6] O. Khaselev , J. A. Turner , Science 1998, 280, 425. 9545218 10.1126/science.280.5362.425

[7] a) Y. Xiao , X. Guo , J. Liu , L. Liu , F. Zhang , C. Li , Chinese J. Catal. 2019, 40, 1339;

[8] a) Z. Wang , L. Wang , Sci. China Mater. 2018, 61, 806;

[9] a) C. V. Reddy , K. R. Reddy , V. V. N. Harish , J. Shim , M. V. Shankar , N. P. Shetti , T. M. Aminabhavi , Int. J. Hydrogen Energy 2020, 45, 7656;

[10] a) D. Wang , R. Huang , W. Liu , D. Sun , Z. Li , ACS Catal. 2014, 4, 4254;

[11] a) D. Sun , W. Liu , M. Qiu , Y. Zhang , Z. Li , Chem. Commun. 2015, 51, 2056; 10.1039/c4cc09407g 25532612

[12] a) Z. Wang , H. Huang , S. A. Monny , M. Xiao , L. Wang , J. Chem. Phys. 2020, 153, 024706; 32668936 10.1063/5.0010722

[13] M. Xiao , Z. Wang , M. Lyu , B. Luo , S. Wang , G. Liu , H. M. Cheng , L. Wang , Adv. Mater. 2019, 31, 1801369. 10.1002/adma.201801369 30125390

[14] D. Kong , Y. Zheng , M. Kobielusz , Y. Wang , Z. M. Bai , W. Macyk , X. C. Wang , J. W. Tang , Mater. Today 2018, 21, 897.

[15] a) D. Shi , R. Zheng , C. S. Liu , D. M. Chen , J. Zhao , M. Du , Inorg. Chem. 2019, 58, 7229; 30994335 10.1021/acs.inorgchem.9b00206

[16] a) T. Wu , X. Liu , Y. Liu , M. Cheng , Z. Liu , G. Zeng , B. Shao , Q. Liang , W. Zhang , Q. He , W. Zhang , Coord. Chem. Rev. 2020, 403, 213097;

[17] a) M. Chang , Y. Zhao , Q. Yang , D. Liu , ACS Omega 2019, 4, 14511; 31528805 10.1021/acsomega.9b01740 PMC6740180

[18] a) A. Munir , K. S. Joya , T. Ul Haq , N. U. Babar , S. Z. Hussain , A. Qurashi , N. Ullah , I. Hussain , ChemSusChem 2019, 12, 1517; 30485695 10.1002/cssc.201802069

[19] a) Q. Mu , W. Zhu , X. Li , C. Zhang , Y. Su , Y. Lian , P. Qi , Z. Deng , D. Zhang , S. Wang , X. Zhu , Y. Peng , Appl. Catal. B 2020, 262, 118144;

[20] Y. Su , Z. Zhang , H. Liu , Y. Wang , Appl. Catal. B 2017, 200, 448.

[21] a) B. Xu , H. Zhang , H. Mei , D. Sun , Coord. Chem. Rev. 2020, 420;

[22] H. Liu , J. Zhang , D. Ao , Appl. Catal. B 2018, 221, 433.

[23] H. Song , Z. Sun , Y. Xu , Y. Han , J. Xu , J. Wu , T. Sun , H. Meng , X. Zhang , Sep. Purif. Technol. 2019, 228, 115764.

[24] P. Buckley , Energy Environ. Sci. 2009, 2009, 344.

[25] a) Y. Liu , D. Huang , M. Cheng , Z. Liu , C. Lai , C. Zhang , C. Zhou , W. Xiong , L. Qin , B. Shao , Q. Liang , Coord. Chem. Rev. 2020, 409, 213220;

[26] J. Shi , J. Dong , S. Lv , Y. Xu , L. Zhu , J. Xiao , X. Xu , H. Wu , D. Li , Y. Luo , Appl. Phys. Lett. 2014, 104, 063901.

[27] X. Li , Q.-L. Zhu , Energy Chem. 2020, 2, 100033.

[28] C. H. Hendon , D. Tiana , M. Fontecave , C. Sanchez , L. D’Arras , C. Sassoye , L. Rozes , C. Mellot-Draznieks , A. Walsh , J. Am. Chem. Soc. 2013, 135, 10942. 23841821 10.1021/ja405350u

[29] S. Mao , J.-W. Shi , G. Sun , Y. Zhang , X. Ji , Y. Lv , B. Wang , Y. Xu , Y. Cheng , Chem. Eng. J. 2021, 404, 126533.

[30] Y.-P. Yuan , L.-S. Yin , S.-W. Cao , G.-S. Xu , C.-H. Li , C. Xue , Appl. Catal. B 2015, 168–169, 572.

[31] L. Y. Wu , Y. F. Mu , X. X. Guo , W. Zhang , Z. M. Zhang , M. Zhang , T. B. Lu , Angew. Chem. Int. Ed. 2019, 58, 9491. 10.1002/anie.201904537 31066965

[32] S. Saha , G. Das , J. Thote , R. Banerjee , J. Am. Chem. Soc. 2014, 136, 14845. 25279940 10.1021/ja509019k

[33] L. Shen , M. Luo , Y. Liu , R. Liang , F. Jing , L. Wu , Appl. Catal. B 2015, 166–167, 445.

[34] X.-B. Meng , J.-L. Sheng , H.-L. Tang , X.-J. Sun , H. Dong , F.-M. Zhang , Appl. Catal. B: Environ. 2019, 244, 340.

[35] X. Hao , Z. Jin , H. Yang , G. Lu , Y. Bi , Appl. Catal. B 2017, 210, 45.

[36] R. Wang , L. Gu , J. Zhou , X. Liu , F. Teng , C. Li , Y. Shen , Y. Yuan , Adv. Mater. Interfaces 2015, 2, 1500037.

[37] K. Li , Y. Zhang , Y. Z. Lin , K. Wang , F. T. Liu , ACS Appl. Mater. Interfaces 2019, 11, 28918. 31333019 10.1021/acsami.9b09312

[38] M. Aleksandrzak , D. Baranowska , T. Kedzierski , K. Sielicki , S. Zhang , M. Biegun , E. Mijowska , Appl. Catal. B Environ. 2019, 257, 117906.

[39] a) L. Sun , Y. Yuan , F. Wang , Y. Zhao , W. Zhan , X. Han , Nano Energy 2020, 74, 104909;

[40] L. Shi , D. Benetti , F. Li , Q. Wei , F. Rosei , Appl. Catal. B 2020, 263, 118317.

[41] Z. Jiao , J. Zheng , C. Feng , Z. Wang , X. Wang , G. Lu , Y. Bi , ChemSusChem 2016, 9, 2824. 27572550 10.1002/cssc.201600761

[42] Z. Peng , S. C. Abbas , J. Lv , R. Yang , M. Wu , Y. Wang , Int. J. Hydrogen Energy 2019, 44, 2446.

[43] a) M. Rad , S. Dehghanpour , RSC Adv. 2016, 6, 61784;

[44] J. Zhou , A. Zhou , L. Shu , M.-C. Liu , Y. Dou , J.-R. Li , Appl. Catal. B 2018, 226, 421.

[45] W. Zhang , R. Li , X. Zhao , Z. Chen , A. W. Law , K. Zhou , ChemSusChem 2018, 11, 2710. 29975458 10.1002/cssc.201801162

[46] S. Zhou , P. Yue , J. Huang , L. Wang , H. She , Q. Wang , Chem. Eng. J. 2019, 371, 885.

[47] J. B. Pan , B. H. Wang , J. B. Wang , H. Z. Ding , W. Zhou , X. Liu , J. R. Zhang , S. Shen , J. K. Guo , L. Chen , C. T. Au , L. L. Jiang , S. F. Yin , Angew. Chem. Int. Ed. 2020, 59, 2.

[48] P. P. Bag , X.-S. Wang , P. Sahoo , J. Xiong , R. Cao , Catal. Sci. Technol. 2017, 7, 5113.

[49] R. Lin , L. Shen , Z. Ren , W. Wu , Y. Tan , H. Fu , J. Zhang , L. Wu , Chem. Commun. 2014, 50, 8533. 10.1039/c4cc01776e 24949823

[50] Y.-J. Dong , J.-F. Liao , Z.-C. Kong , Y.-F. Xu , Z.-J. Chen , H.-Y. Chen , D.-B. Kuang , D. Fenske , C.-Y. Su , Appl. Catal. B: Environ. 2018, 237, 9.

[51] S. Zhou , K. Chen , J. Huang , L. Wang , M. Zhang , B. Bai , H. Liu , Q. Wang , Appl. Catal. B: Environ. 2020, 266, 118513.

[52] D. Xu , T. Xia , W. Fan , H. Bai , J. Ding , B. Mao , W. Shi , Appl. Surf. Sci. 2019, 491, 497.

[53] W. Cui , J. Shang , H. Bai , J. Hu , D. Xu , J. Ding , W. Fan , W. Shi , Chem. Eng. J. 2020, 388, 124206.

[54] C.-H. Li , C.-L. Huang , X.-F. Chuah , D. Senthil Raja , C.-T. Hsieh , S.-Y. Lu , Chem. Eng. J. 2019, 361, 660.

[55] Y. Dou , J. Zhou , A. Zhou , J.-R. Li , Z. Nie , J. Mater. Chem. A 2017, 5, 19491.

